# Building momentum for malaria vaccine research and development: key considerations

**DOI:** 10.1186/s12936-020-03491-3

**Published:** 2020-11-23

**Authors:** Chetan E. Chitnis, David Schellenberg, Johan Vekemans, Edwin J. Asturias, Philip Bejon, Katharine A. Collins, Brendan S. Crabb, Socrates Herrera, Miriam Laufer, N. Regina Rabinovich, Meta Roestenberg, Adelaide Shearley, Halidou Tinto, Marian Wentworth, Kate O’Brien, Pedro Alonso

**Affiliations:** 1 Institut Pasteur, Paris, France; 2 World Health Organization, Geneva, Switzerland; 3 University of Colorado School of Medicine and Colorado School of Public Health, Denver, USA; 4 KEMRI-Wellcome Trust Research Programme, Kilifi, Kenya; 5 Department of Medical Microbiology, Radboud University Medical Center, Nijmegen, The Netherlands; 6 Burnet Institute, Melbourne, Australia; 7 Consorcio Para La Investigacion Cientifica, Cali, Colombia; 8 University of Maryland School of Medicine, Baltimore, USA; 9 IS Global, Barcelona, Spain; 10 Harvard TH Chan School of Public Health, Boston, USA; 11 Leiden University Medical Center, Leiden, The Netherlands; 12 John Snow Inc, Research & Training Institute, Boston, USA; 13 Institut de Recherche en Sciences de La Santé, Ouagadougou, Burkina Faso; 14 Management Sciences for Health, Arlington, USA

**Keywords:** Malaria, Vaccine, Control, Elimination, Research and development

## Abstract

To maintain momentum towards improved malaria control and elimination, a vaccine would be a key addition to the intervention toolkit. Two approaches are recommended: (1) promote the development and short to medium term deployment of first generation vaccine candidates and (2) support innovation and discovery to identify and develop highly effective, long-lasting and affordable next generation malaria vaccines.

## Background

In what is a truly great public health success story, expanded efforts to control and eliminate malaria have effectively halved malaria incidence and mortality since 2000. Several million lives have been saved in that time and a number of previously endemic countries in Asia, South and Central America and Africa have been formally declared malaria free.

This astonishing success has been achieved with a limited toolkit, largely comprising methods to prevent transmission by the mosquito vector through the use of insecticide-treated bed nets and indoor residual spraying, the use of chemoprevention in specific, vulnerable groups, and effective chemotherapy following rapid point-of-care diagnosis. Current vector control and effective anti-malarial treatment strategies represent significant success in both product development and implementation science.

However, progress in areas with high transmission has slowed and further reduction in malaria incidence and deaths has stalled in recent years. The 2018 and 2019, World Health Organization (WHO) World Malaria Reports documented a global increase in the number of malaria cases. Despite some countries achieving elimination, malaria increased in both the 10 most highly burdened countries and 11 of the 21 countries earmarked for elimination by 2020 [1].

A number of daunting realities impact on the potential for substantial further progress. These include: (1) malaria remains a staggeringly large human health problem with 1,200 malaria deaths every day, (2) longitudinal tracking of the effective implementation of existing tools show imperfect outcomes and suggests that existing tools may be insufficient to control malaria in high-transmission settings, no matter how well they are applied, (3) shifts in climate, population growth and movement, and changes in the location and species of vector, threaten to introduce malaria into new settings (for example, greater urban transmission in Africa by *Anopheles stephensi*), (4) problems achieving high coverage of current interventions are exacerbated by the emergence of vectors resistant to insecticides, parasites resistant to first-line treatment and parasite strains that evade diagnosis, (5) lessons from the 1970s and our knowledge of parasite biology and ecology tell us that resurgence can be rapid and devastating if public health measures fail or are not maintained, and (6) the COVID-19 pandemic has exposed the vulnerability of global supply chains and the health systems in many malaria endemic settings. Hard won gains can rapidly be lost.

## Main text

New interventions are needed to reignite the fight against malaria. As for other infectious diseases, vaccines have the potential to impact burden in a cost-effective way and may, in the long term, contribute to the goal of malaria eradication. The feasibility of vaccine-induced protection against malaria has been demonstrated [2], but the development of malaria vaccines requires the vigorous and sustained engagement of many stakeholders. Recent advances in the understanding of malaria parasite biology, vaccinology and passive immunization approaches, suggest that the next advance in malaria vaccines is within reach─but only with sustained research and development efforts.

The WHO reconvened the Malaria Vaccine Advisory Committee (MALVAC) in 2019, and organized a stakeholder consultation about the state-of-the-art in malaria vaccine development [Vekemans et al. pers. commun.]. MALVAC’s mandate is to provide guidance on research priorities for the development of new malaria vaccines. Detailed WHO perspectives on the medical need and research priorities in malaria vaccine R&D will emerge over the next 12–24 months, but consultations and MALVAC discussions led to the recognition of the need to advance in parallel two distinct strategies:

1. To support continued engagement to ensure the availability of 1st and 2nd generation vaccine candidates with moderate efficacy, that show potential for widespread use in the next 3–10 years.
2. To support innovation and stimulate the discovery of next generation, highly protective and long-lasting malaria vaccines; for this to succeed, identifying efficient and cost-effective clinical development, financing and regulatory pathways will be key. Lessons can no doubt be learnt from the accelerated development pathways and approaches being developed for COVID-19 vaccines.

### 1st Generation Vaccines with partial protection—an important addition to the intervention toolkit

The most advanced malaria vaccine is RTS,S/AS01, developed by Glaxo Smith Kline with support from the Bill and Melinda Gates Foundation, the Walter Reed Army Institute of Research and PATH, and the collaboration of a large number of African and other international research institutions. RTS,S/AS01 targets *Plasmodium falciparum* sporozoites and demonstrated an efficacy of 39% over 4 years against malaria incidence in Phase III trials in African children aged 5–17 months at the time of dose 1 [3]. This moderate efficacy, documented in the context of high mosquito net use and similar to the level of protection afforded by well-implemented vector control, is potentially valuable to complement existing strategies for the reduction of malaria disease and death among young children in endemic areas. RTS,S/AS01 pilot implementation is ongoing in three malaria endemic countries—Ghana, Malawi and Kenya [4]. In addition to consolidating the vaccine’s safety profile, the pilot implementation will generate data on its survival impact and test the feasibility of delivering the four-dose RTS,S/AS01 regimen under routine conditions. Results of the implementation studies are keenly awaited and will be used to guide policy recommendations on the roll out of RTS,S/AS01 in malaria endemic countries.

RTS,S/AS01 has demonstrated the feasibility of developing a malaria vaccine and has laid down a clinical development path for future vaccines. Its use in programmatic contexts will inform our understanding of the potential value of malaria vaccines in combination with other tools for malaria control and elimination.

In addition to RTS,S/AS01, R21/Matrix-M, an RTS,S-like vaccine, is one of several potential second generation vaccines and is currently being tested for efficacy in the field. Notwithstanding enormous technical and practical challenges, the radiation-attenuated sporozoite vaccine, PfSPZ, has undergone extensive testing including in endemic African countries. Although high efficacy has been demonstrated in adults under experimental challenge conditions, efficacy in naturally exposed children is considerably lower, warranting further improvements. Progress is also being made through the evaluation of Rh5, a promising *P. falciparum* blood stage vaccine candidate, although it will be necessary to achieve higher rates of growth inhibition for such vaccines to yield clinically relevant efficacy.

The evaluation of sexual-stage candidates continues, and new tools to test vaccines designed to interrupt man-to mosquito transmission are being developed. Subunit vaccines that combine multiple antigens from the pre-erythrocytic and blood stages could synergize immune responses and yield higher efficacy. The addition of sexual-stage antigens to these vaccines could potentially enhance their impact on malaria transmission [2]. Continued investment in the development of these approaches is warranted given the progress to date and the scale of their potential impact on public health. In addition to subunit vaccines, innovations in the development of whole organism attenuated sporozoite vaccines are needed to develop formulations and delivery strategies that facilitate programmatic implementation in endemic countries.

### Future malaria vaccines–towards highly efficacious, long-lasting vaccines and a more streamlined development pathway

Malaria vaccines that confer long-term, robust protection, that are inexpensive and relatively simple to deploy, are not on the short-term horizon. To accelerate progress in the development of such vaccines, a deliberate strategic pivot to fundamental discovery science is needed. Breakthrough science, with possibly unconventional approaches, will be required to meet these ambitious goals [5].

Decoding of the malaria parasite genome together with functional studies using molecular genetic tools, whole genome approaches as well as classical biochemistry and cell biology, are helping unravel the complex biology of the malaria parasite. Advances in understanding how malaria parasites interact with the human host and its immune system should enable new strategies to target the parasite at different stages with novel vaccine approaches. Advances in our understanding of basic human immunology and powerful new tools that enable dissection of immune responses at a systems level need to be brought to bear on malaria. Other advances such as structural vaccinology can provide unique insights into the molecular basis of protective antibody responses that could lead to therapeutic or prophylactic monoclonal antibodies and inform optimization of vaccine antigens to achieve higher efficacy.

## Conclusion

The development of vaccines against parasitic diseases is complex and difficult due to the long history of co-evolution of parasites with their hosts. Malaria vaccines are currently envisioned as complementary tools to be added to the core package of interventions. However, the progress made in understanding malaria parasite biology and pathogenesis, as well as both basic and technological advances in human immunology and vaccinology, means the time is right to attempt the development of malaria vaccines with high efficacy. It is time to deepen and expand our ambitions at all levels, basic and translational, to develop future malaria vaccines that are game changers in efforts to eliminate malaria and create a pathway for other parasitic diseases. A highly efficacious malaria vaccine remains an ambitious target, but with commitment of necessary resources, it is more within reach today than ever before.

## Data Availability

Not applicable.

## Abbreviations

WHO: World Health Organization
MALVAC: Malaria Vaccine Advisory Committee
